#  Tendência temporal de atividade física em adolescentes brasileiros: análise da *Pesquisa Nacional de Saúde do Escolar* de 2009 a 2019 

**DOI:** 10.1590/0102-311XPT063423

**Published:** 2023-11-13

**Authors:** Carlos Alex Martins Soares, Otávio Amaral de Andrade Leão, Matheus Pintanel Freitas, Pedro Curi Hallal, Mário Bernardes Wagner

**Affiliations:** 1 Programa de Pós-graduação em Saúde da Criança e do Adolescente, Universidade Federal do Rio Grande do Sul, Porto Alegre, Brasil.; 2 Programa de Pós-graduação em Educação Física, Universidade Federal de Pelotas, Pelotas, Brasil.; 3 Programa de Pós-graduação em Epidemiologia, Universidade Federal de Pelotas, Pelotas, Brasil.

**Keywords:** Adolescente, Atividade Física, Fatores de Risco, Estudos de Séries Temporais, Adolescent, Physical Activity, Risk Factors, Time Series Studies, Adolescente, Actividad Física, Factores de Riesgo, Estudios de Series Temporales

## Abstract

O objetivo do estudo foi analisar a tendência de atividade física dos escolares brasileiros e as associações com variáveis demográficas, socioeconômicas e comportamentais, por meio da *Pesquisa Nacional de Saúde do Escolar* (PeNSE) em suas quatro edições - 2009, 2012, 2015 e 2019. Foram usados dados dos escolares (13-17 anos) participantes das quatro edições da PeNSE (n = 392.922). Descrevemos o percentual de ativos, a média e valores percentuais da atividade física de intensidade moderada à vigorosa em minutos/semana. A regressão de Poisson foi ajustada para sexo, idade, cor da pele, escore de bens e comportamento sedentário (≥ 2 horas/dia para assistir TV e ≥ 3 horas/dia de tempo sentado). Como limitação, a amostra da PeNSE/2009 refere-se apenas às capitais brasileiras. O percentual de ativos reduziu de 43,1% em 2009 para 18,2% em 2019. A média em atividade física de intensidade moderada à vigorosa da PeNSE/2009 (média = 318,4 minutos/semana; IC95%: 313,4-323,4) reduziu 50% em 2019. Na educação física, a média semanal em atividade física de intensidade moderada à vigorosa das meninas foi menor que 50 minutos, ao passo que a dos meninos foi maior que 60 minutos, nas quatro edições da PeNSE. Ainda, 22,7% das meninas relataram (PeNSE/2019) não ter tido aulas de educação física, enquanto o mesmo é relatado por 19,7% dos meninos. O comportamento sedentário sofreu redução no hábito de assistir TV, porém o tempo sentado aumentou de 50,1% (IC95%: 48,9-51,3) para 54% (IC95%: 53,1-54,9) entre a PeNSE/2009 e a PeNSE/2019. Como consequência da queda nos níveis de atividade física, são necessárias políticas públicas que promovam a atividade física, como aumentar as aulas de educação física na escola para, no mínimo, três vezes por semana.

## Abstract (en)

This study aimed to analyze the physical activity trend of Brazilian schoolchildren and the associations with demographic, socioeconomic, and behavioral variables by using the *Brazilian National Survey of School Health* (PeNSE) in its four editions - 2009, 2012, 2015, and 2019. Data from students (13-17 years old) participating in the four editions of the PeNSE (n = 392,922) were used. We describe the percentage of active, mean, and percentile values of moderate to vigorous intensity physical activity in minutes/week. Poisson’s regression was adjusted for gender, age, skin color, goods score, and sedentary behavior (≥ 2 hours/day watching TV and ≥ 3 hours/day sitting time). As a limitation, the PeNSE/2009 sample refers only to the Brazilian capital cities. The percentage of active students decreased from 43.1% in 2009 to 18.2% in 2019. The mean moderate to vigorous intensity physical activity of PeNSE/2009 (mean = 318.4 minutes/week; 95%CI: 313.4-323.4) decreased 50% in 2019. In physical education, the weekly average in moderate to vigorous intensity physical activity of girls is less than 50 minutes and boys is greater than 60 minutes in the four editions of PeNSE, also 22.7% of girls reported (PeNSE/2019) not having taken physical education classes whereas the same thing is reported by 19.7% of boys. Sedentary behavior reduced regarding time watching TV, but sitting time increased by 50.1% (95%CI: 48.9-51.3) and 54% (95%CI: 53.1-54.9) between PeNSE/2009 and PeNSE/2019. As a consequence of the drop in physical activity levels, public policies that promote physical activity are necessary, including increasing physical education classes at school to at least three times a week.

## Resumen

El objetivo de este estudio fue analizar la tendencia de la actividad física del alumnado brasileño y sus asociaciones con las variables demográficas, socioeconómicas y de comportamiento mediante la *Encuesta Nacional de Salud del Escolar* (PeNSE) en sus cuatro ediciones (de 2009, 2012, 2015 y 2019). Se utilizaron los datos del alumnado (13-17 años) que participó en las cuatro ediciones de la PeNSE (n = 392.922). Se describieron el porcentaje de valores activos, la media y los valores percentiles de actividad física de intensidad moderada a vigorosa en minutos/semana. La regresión de Poisson se ajustó según sexo, edad, color de la piel, puntuación de activos y comportamiento sedentario (≥ 2 horas/día para ver televisión y ≥ 3 horas/día sentado). Como limitación, la muestra de la PeNSE/2009 se refiere únicamente a las capitales brasileñas. El porcentaje de activos disminuyó del 43,1% en 2009 al 18,2% en 2019. La media de actividad física de intensidad moderada a vigorosa de la PeNSE/2009 (media = 318,4 minutos/semana; IC95%: 313,4-323,4) tuvo una reducción de un 50% en 2019. En educación física, el promedio semanal en actividad física de intensidad moderada a vigorosa de las niñas fue menos de 50 minutos, y el de los niños llegó a 60 minutos en las cuatro ediciones de la PeNSE, además, el 22,7% de las niñas y el 19,7% de los niños (PeNSE/2019) informaron no haber tomado clases de educación física. Hubo una disminución en el comportamiento sedentario de ver televisión, pero el tiempo sentado aumentó del 50,1% (IC95%: 48,9-51,3) al 54% (IC95%: 53,1-54,9) entre la PeNSE/2009 y la PeNSE/2019. El descenso de los niveles de actividad física lleva a la necesidad de desarrollar políticas públicas que promuevan la actividad física, incluido el incremento de las clases de educación física en las escuelas al menos tres veces por semana.

## Introdução

As doenças crônicas não transmissíveis (DCNT) se desenvolvem silenciosamente ao longo da vida, têm desfechos precoces (entre 30 e 69 anos) e são consequências de uma associação de fatores genéticos, fisiológicos, ambientais e comportamentais. Cerca de um quarto dos brasileiros tem pelo menos uma DCNT ^1^^,^^2^. Destacam-se doenças cardiovasculares, cânceres, doenças respiratórias crônicas e diabetes tipo 2, que foram responsáveis por 71% dos óbitos e por 85% das mortes prematuras no mundo em 2016 ^3^. No Brasil, causaram 54,7% dos óbitos em 2019 e, dessas mortes, 41,8% foram prematuras, sendo que em 2000 havia ocorrido 47,4% de óbitos precoces ^4^. A prevenção para esse tipo de doenças ocorre a partir do monitoramento e da vigilância, seguidos pelo desenvolvimento de políticas públicas que contribuam para alcançar as metas da Organização Mundial da Saúde (OMS) de reduzir a inatividade física em 10% até 2025 e em 15% até 2030 ^5^^,^^6^.

Em 2016, 28% dos adultos (18+ anos) não atenderam às recomendações da OMS para atividade física, ou seja, não cumpriram de 150 a 300 minutos por semana de atividade física com intensidade moderada à vigorosa. A prevalência de inatividade física é mais do que o dobro em países de alta renda em relação aos de média e baixa rendas, as mulheres são menos ativas na maioria dos países membro da OMS e, nos últimos 15 anos, os níveis de inatividade física não diminuíram ^7^. Além disso, o nível de atividade física costuma declinar durante a adolescência ^8^. Relatórios da *Pesquisa Nacional de Saúde do Escolar* (PeNSE) ^9^^,^^10^ mostram que o percentual de adolescentes que não atingiram as recomendações para atividade física cresceu de 56,9% na PeNSE/2009 para 71,8% da PeNSE/2012. Certamente, entre as justificativas para não atender às diferentes recomendações, está a tendência mundial de baixos níveis de atividade física e altos níveis de comportamento sedentário na infância e na adolescência ^11^.

Enquanto isso, o Brasil caminha lentamente no monitoramento e vigilância da saúde dos adolescentes. Observa-se que estudos semelhantes em outros países são realizados há mais de quatro décadas, como o transnacional *Health Behaviour School-based Children* (HBSC; Estudo Comportamental de Saúde de Escolares) em mais de 40 países europeus, o *Global School-based Student Health Survey* (GSSH; Pesquisa Global de Saúde dos Escolares) da OMS e o *Youth Risk Behavior Surveillance System* (YRBSS; Sistema de Vigilância de Comportamentos de Risco Juvenil) do Centro de Controle e Prevenção de Doenças dos Estados Unidos (CDC). Há um alerta nesses estudos em relação à atividade física dos adolescentes, pois a grande maioria dos jovens não está atingindo as recomendações para atividade física semanal, como explicitam o HBSC/2016 (82%) ^12^ e a PeNSE/2019 (81,9%) ^10^.

Em nível mundial, as aferições da atividade física em adolescentes têm como base o ponto de corte para atividade física recomendado pela OMS ^5^, ou seja, atividade física de intensidade moderada à vigorosa, frequência diária e duração mínima de 60 minutos por sessão ^7^^,^^13^. Essas recomendações foram reafirmadas e atualizadas em propostas posteriores, tanto pela OMS ^13^ quanto pelo Departamento de Saúde e Serviços Humanos dos Estados Unidos (DHHS) ^14^ - e, inclusive, pelo Ministério da Saúde brasileiro ^15^.

Assim, o acompanhamento do nível de atividade física dos adolescentes insere-se na vigilância em saúde do Ministério da Saúde, ou seja, na necessidade de identificar e acompanhar os principais indicadores de saúde e a probabilidade de o adulto desenvolver alguma DCNT, pois a prática de atividade física nessa faixa etária é preditora do comportamento fisicamente ativo ou inativo na idade adulta ^16^. Se a aquisição e incorporação da atividade física como hábito saudável ocorre pela influência dos pais, dos pares e do ambiente escolar e deve ser desenvolvida a longo prazo ^17^, consequentemente, a vigilância do nível de atividade física dos adolescentes contribui para um planejamento em saúde abrangente, elaborado e direcionado à promoção de ações que visem mitigar os efeitos negativos da inatividade física.

Nesse cenário, a relevância e a análise dos dados da PeNSE permitirão compreender a tendência da atividade física entre adolescentes na última década, projetar o futuro, elencar ações necessárias para reduzir o desenvolvimento precoce dos fatores de risco e o subsequente surgimento de DCNT. Portanto, nosso objetivo é analisar a tendência de atividade física dos escolares brasileiros e as associações com variáveis demográficas, socioeconômicas e comportamentais por meio da PeNSE em suas quatro edições - 2009, 2012, 2015 e 2019.

## Métodos

### Delineamento

Esta é uma pesquisa epidemiológica, transversal, de base escolar e composta por análises ecológicas ^18^, pois utiliza dados governamentais coletados pelo Instituto Brasileiro de Geografia e Estatística (IBGE) em quatro edições da PeNSE (2009, 2012, 2015 e 2019), a partir da proposição de vigilância em saúde do adolescente realizada pela Secretaria de Vigilância em Saúde do Ministério da Saúde.

### População-alvo

A população-alvo da PeNSE são adolescentes, de ambos os sexos, matriculados e frequentes nos Ensinos Fundamental e Médio de escolas públicas e privadas situadas nas zonas urbanas e rurais de todo o território brasileiro. A série e a idade foram definidas por estarem relacionadas, respectivamente, com o mínimo de escolarização e a fase em que os indivíduos já têm autonomia necessária, preconizada pela OMS, para responder a um questionário autoaplicável.

Na primeira edição da PeNSE (2009), a coleta de dados foi restrita às 27 capitais brasileiras, com N = 618.553 e uma amostra de 61.434 escolares. Em 2012, foi mantida a coleta de dados nas capitais e foram incluídas cidades do interior, agrupadas em cada uma das cinco grandes regiões político-administrativas do país (Norte, Nordeste, Centro-oeste, Sudeste e Sul), elevando a população-alvo (N = 3.153.314) e obtendo amostra de 106.480 escolares.

Em 2015, o plano amostral sofreu adaptações e foram desenvolvidas duas amostras. Neste estudo, utilizamos a amostra 1, composta pelas 27 capitais e pelos municípios do interior, agrupados por Unidades da Federação e abrangendo todo o território brasileiro. Assim, foram criados 53 estratos geográficos, com a população-alvo estimada em 2.630.835 de escolares e a amostra final com 100.110 escolares.

Finalmente, em 2019, a partir da população-alvo, estimada em 11.851.941 de escolares na faixa etária de 13 a 17 anos, matriculados do 7º ao 9º do Ensino Fundamental e do 1º ao 3º ano do Ensino Médio, a amostra totalizou 124.898 escolares.

### Amostra

A amostra deste estudo é o conjunto das quatro edições da PeNSE, cujas amostras foram aleatórias, probabilísticas, estratificadas e dimensionadas para estimar parâmetros populacionais (proporções ou prevalências), representativas para terem validade interna, externa e significância estatística. A amostra foi a estimativa da proporção populacional, calculada para fornecer estimativas de proporções de algumas características de interesse, em cada um dos estratos geográficos, utilizando-se erro amostral máximo de 3%, nível de 95% de confiança, prevalência de 50%, pois para proporções desse valor a variância dos estimadores amostrais é máxima.

Assim, este estudo é baseado em dados públicos e os projetos originais foram submetidos e aprovados pela Comissão Nacional de Ética em Pesquisa (CONEP). Nossa amostra é composta por 392.922 escolares, de ambos os sexos, da faixa etária de 13 a 17 anos e oriundos da PeNSE/2009 (n = 61.434), PeNSE/2012 (n = 106.480), PeNSE/2015 (n = 100.110) e PeNSE/2019 (n = 124.898).

Desde 2009, o Ministério da Saúde, sob a liderança da Secretaria de Vigilância em Saúde, tem realizado a PeNSE, em parceria com o IBGE e o Instituto Nacional de Estudos e Pesquisas Educacionais Anísio Teixeira (INEP) do Ministério da Educação, além do suporte das Secretarias Estaduais e Municipais de Saúde e de Educação dos estados e municípios brasileiros. Nesse período, por meio de estudos epidemiológicos transversais, de base escolar, quatro edições da PeNSE (2009, 2012, 2015 e 2019) coletaram dados de adolescentes dos Ensinos Fundamental e Médio de escolas públicas e privadas.

### Variáveis do estudo

A variável dependente foi a atividade física acumulada. Esse desfecho foi obtido por autorrelato, utilizando informações de três domínios: deslocamentos casa-escola-casa, atividade física nas aulas de educação física e atividade física de lazer - consideram-se as atividades realizadas na escola, mas distintas da aula regular de educação física e atividades físicas diversas realizadas em outros espaços (clubes, associações, praças etc.). Com essas informações, foi realizada a multiplicação da frequência (dias) pela duração (tempo de prática diária) da atividade física em cada domínio, seguida pela soma dos produtos de cada domínio, resultando na atividade física acumulada.

Posteriormente, a atividade física acumulada foi dicotomizada em atingir (≥ 300 minutos/semana) e não atingir (< 300 minutos/semana) as recomendações para atividade física. A categoria “atingir” foi denominada “percentual de ativos fisicamente” e teve seu intervalo de 95% de confiança (IC95%) reportado para cada edição da PeNSE, distribuição geográfica (Brasil, capitais e interior) e estratificado por sexo, idade e escore de bens (Tabela 1). Destaca-se que o tempo mínimo recomendado de atividade física para jovens é de 60 minutos por dia de atividade física de intensidade moderada à vigorosa ^19^^,^^20^, frequência diária ^7^^,^^13^^,^^14^, sendo que em três dias devem ser desenvolvidas atividade de fortalecimento osteomuscular ^14^.

**Tabela 1:** Percentual de ativos fisicamente segundo distribuição geográfica, sexo, idade e escore de bens e serviços da *Pesquisa Nacional de Saúde do Escolar* (PeNSE) de 2009, 2012, 2015 e 2019.

Variáveis	PeNSE							
	2009 (n = 61.434)		2012 (n = 106.480)		2015 (n = 100.110)		2019 (n = 124.898)	
	%	IC95%	%	IC95%	%	IC95%	%	IC95%
Brasil								
Sexo								
Masculino	-	-	40,6	39,6-41,6	44,1	43,1-45,2	26,7	25,9-27,6
Feminino	-	-	22,6	22,1-23,2	25,5	24,6-26,4	9,3	8,8-9,8
Ambos os sexos			31,2	30,5-32,0	34,5	33,8-35,3	18,0	17,5-18,6
Idade (anos)								
13-15	-	-	31,5	31,0-32,1	34,5	33,7-35,3	18,1	17,5-18,8
16-17	-	-	29,0	26,6-31,5	34,7	33,1-36,3	17,8	16,9-18,7
Escore de bens e serviços *								
≤ 1	-	-	22,3	19,3-25,6	27,3	25,3-29,5	15,8	14,1-17,7
2	-	-	27,2	25,7-28,7	30,2	28,7-31,7	17,5	16,0-19,0
3	-	-	31,2	29,8-32,7	33,8	32,6-35,0	16,6	15,8-17,4
4	-	-	34,7	33,8-35,5	36,6	35,6-37,5	19,3	18,5-20,1
Capitais								
Sexo								
Masculino	55,8	54,7-56,8	43,6	42,2-45,0	45,6	44,3-47,0	26,5	25,4-27,6
Feminino	31,6	30,5-32,8	25,0	24,0-26,0	27,1	26,0-28,3	9,8	9,1-10,6
Ambos os sexos	43,1	42,2-43,9	34,1	33,2-35,0	36,2	35,2-37,3	18,2	17,5-19,0
Idade (anos)								
13-15	43,1	42,2-44,1	34,2	33,2-35,1	35,8	34,6-36,9	18,8	17,8-19,8
16-17	42,6	40,5-44,7	33,5	31,6-35,5	41,5	39,4-43,7	17,2	16,2-18,3
Escore de bens e serviços *								
≤ 1	41,2	39,0-43,4	27,2	24,8-29,7	34,3	30,5-38,4	18,1	15,2-21,4
2	39,4	37,7-41,2	29,9	28,2-31,7	38,3	35,7-41,0	16,7	15,0-18,6
3	43,7	41,8-45,6	33,6	31,8-35,5	35,2	33,4-37,1	17,1	16,0-18,2
4	45,8	44,4-47,1	35,6	34,6-36,6	36,3	35,2-37,5	19,0	18,2-20,0
Interior								
Sexo								
Masculino	-	-	39,7	38,1-41,4	43,7	42,4-45,0	26,8	25,7-27,9
Feminino	-	-	22,0	21,1-22,9	25,0	23,8-26,1	9,1	8,5-9,7
Ambos os sexos	-	-	30,4	29,2-31,6	34,0	33,1-35,0	17,9	17,3-18,6
Idade (anos)								
13-15	-	-	30,8	29,9-31,6	34,1	33,1-35,2	17,9	17,1-18,8
16-17	-	-	27,9	23,8-32,3	33,0	31,2-35,0	18,0	16,8-19,1
Escore de bens e serviços *								
≤ 1	-	-	21,7	17,5-26,6	26,6	24,4-28,9	15,6	13,7-17,7
2	-	-	26,7	24,5-29,0	28,8	27,2-30,5	17,6	15,9-19,5
3	-	-	30,6	28,2-33,1	33,4	32,0-34,9	16,5	15,5-17,5
4	-	-	34,3	32,9-35,7	36,6	35,4-37,9	19,4	18,4-20,5

IC95%: intervalo de 95% de confiança.

* Criado a partir da posse de celular, computador, acesso à Internet e banheiro em casa.

As variáveis independentes são demográficas (etnia, sexo, idade), socioeconômicas (escore de posse de bens e serviços) e comportamentais (comportamento sedentário relacionado ao tempo assistindo televisão e relacionado ao tempo sentado). Com relação ao tempo assistindo televisão, foi considerado excessivo o tempo ≥ 2 horas por dia; e quanto ao tempo sentado, ≥ 3 horas por dia. A idade incluída foi limitada à faixa etária de 13 a 17 anos e o sexo restringiu-se à determinação biológica, masculino ou feminino. Como de praxe, a determinação da etnia/cor da pele foi autodeclarada e seguiu as categorias propostas pelo IBGE (branca, parda, preta, amarela e indígena).

Partiu-se das questões sobre bens e serviços, incluídas na PeNSE, para gerar um escore de bens e serviços, conforme relatório do IBGE. Para o escore, foram consideradas as variáveis de posse de celular (0 = não; 1 = sim), computador ou *notebook* (0 = não; 1 = sim), acesso à Internet em casa (0 = não; 1 = sim) e ter banheiro completo na residência (0 = não; 1 = sim). O escore foi contabilizado como a soma dos itens, sendo “0” um indivíduo que não tem nenhum dos itens e “4” o indivíduo que tem todos os itens.

### Análise estatística

A análise dos dados foi realizada primeiramente mediante a descrição da frequência relativa (Material Suplementar: Material Suplementar https://cadernos.ensp.fiocruz.br/static//arquivo/suppl-e00063423_6796.pdf) das variáveis utilizadas nas quatro edições da PeNSE (2009, 2012, 2015 e 2019). A atividade física foi utilizada para verificar a diferença entre os inquéritos e descrita por meio de: (i) percentual de ativos fisicamente e seus respectivos IC95%, conforme a distribuição geográfica (Brasil, capitais e interior), considerando sexo, idade e escore de bens e serviços (Tabela 1); (ii) médias da atividade física e seus respectivos IC95%, conforme a distribuição geográfica (Brasil, capitais e interior), considerando os domínios da atividade física e o sexo (Tabela 2); e (iii) média geral (ambos os sexos) e dos percentis da atividade física, conforme a distribuição geográfica (Brasil, capitais e interior), considerando os domínios da atividade física e a atividade física acumulada (Tabela 3).

**Tabela 2:** Média em atividade física, expressa em minutos por semana, segundo distribuição geográfica, domínios da atividade física e sexo, conforme *Pesquisa Nacional de Saúde do Escolar* (PeNSE) de 2009, 2012, 2015 e 2019.

Variáveis	PeNSE							
	2009 (n = 61.434)		2012 (n = 106.480)		2015 (n = 100.110)		2019 (n = 124.898)	
	<mml:math><mml:mover accent="true"><mml:mrow><mml:mi>x</mml:mi></mml:mrow><mml:mo>-</mml:mo></mml:mover></mml:math>*	IC95%	<mml:math><mml:mover accent="true"><mml:mrow><mml:mi>x</mml:mi></mml:mrow><mml:mo>-</mml:mo></mml:mover></mml:math>*	IC95%	<mml:math><mml:mover accent="true"><mml:mrow><mml:mi>x</mml:mi></mml:mrow><mml:mo>-</mml:mo></mml:mover></mml:math>*	IC95%	<mml:math><mml:mover accent="true"><mml:mrow><mml:mi>x</mml:mi></mml:mrow><mml:mo>-</mml:mo></mml:mover></mml:math>*	IC95%
Brasil								
Educação física escolar								
Masculino	-	-	62,5	59,0-66,0	61,2	59,5-63,0	60,5	58,8-62,2
Feminino	-	-	41,0	37,2-44,7	41,1	39,6-42,6	36,0	34,4-37,6
Ambos os sexos	-	-	51,3	47,2-55,3	50,9	49,5-52,3	48,4	46,9-49,8
Atividade física em deslocamento ativo								
Masculino	-	-	57,4	56,0-58,7	87,4	83,9-91,0	43,5	41,8-45,2
Feminino	-	-	60,4	58,4-62,3	88,0	84,5-91,4	45,6	43,9-47,4
Ambos os sexos	-	-	58,9	57,3-60,5	87,7	84,6-90,8	44,5	43,1-45,9
Atividade física de lazer **								
Masculino	-	-	160,5	154,5-166,4	154,2	151,2-157,2	167,1	163,8-170,5
Feminino	-	-	86,7	84,8-88,8	78,7	76,2-81,2	97,8	94,9-100,8
Ambos os sexos	-	-	122,0	119,1-124,8	115,4	113,2-117,5	138,5	135,9-141,0
Atividade física acumulada								
Masculino	-	-	280,6	275,6-285,5	302,1	296,8-307,4	207,9	204,2-211,7
Feminino	-	-	188,3	183,6-193,0	207,2	203,0-211,5	110,5	107,7-113,2
Ambos os sexos	-	-	232,4	227,7-237,0	253,3	249,4-257,2	159,2	156,4-162,0
Capitais								
Educação física escolar								
Masculino	67,8	65,2-70,5	68,5	65,9-71,2	62,3	59,4-65,1	63,2	61,0-65,4
Feminino	48,4	45,9-50,9	46,8	44,6-49,0	46,2	43,9-48,5	38,2	36,0-40,3
Ambos os sexos	57,6	55,2-60,0	57,4	55,2-59,7	54,1	51,8-56,4	50,7	48,9-52,5
Atividade física em deslocamento ativo								
Masculino	82,3	78,8-85,7	60,5	58,1-62,9	86,5	82,2-90,7	45,2	43,0-47,3
Feminino	87,9	83,4-92,4	63,3	60,1-66,6	84,1	79,3-88,9	47,1	44,3-49,8
Ambos os sexos	85,2	81,6-88,8	62,0	59,6-64,3	85,3	81,2-89,4	46,1	44,0-48,1
Atividade física de lazer **								
Masculino	253,0	247,2-258,8	167,2	162,4-171,9	159,7	155,3-164,0	171,5	167,1-175,9
Feminino	114,1	109,0-119,1	92,1	88,8-95,3	86,2	82,1-90,2	107,2	103,6-110,7
Ambos os sexos	179,9	175,5-184,3	128,8	125,7-132,0	122,3	118,9-125,6	145,3	142,0-148,7
Atividade física acumulada								
Masculino	397,5	390,7-404,3	296,3	289,9-302,7	307,4	300,2-314,5	209,7	205,1-214,3
Feminino	247,0	241,1-252,9	202,4	197,7-207,1	215,9	210,8-221,1	114,9	110,8-119,0
Ambos os sexos	318,4	313,4-323,4	248,4	243,9-252,8	260,9	255,7-266,1	162,7	159,1-166,2
Interior								
Educação física escolar								
Masculino	-	-	60,7	54,8-66,5	60,9	58,8-63,0	59,8	57,7-61,9
Feminino	-	-	39,3	33,3-45,4	39,6	37,8-41,3	35,4	33,4-37,3
Ambos os sexos	-	-	49,4	42,8-56,1	49,9	48,2-51,6	47,7	45,9-49,4
Atividade física em deslocamento ativo								
Masculino	-	-	56,4	54,3-58,5	87,7	83,3-92,2	43,0	40,9-45,1
Feminino	-	-	59,5	56,4-62,6	89,1	84,9-93,4	45,2	43,1-47,4
Ambos os sexos	-	-	58,0	55,5-60,6	88,5	84,6-92,3	44,1	42,4-45,8
Atividade física de lazer **								
Masculino	-	-	158,4	148,5-168,4	152,5	148,7-156,3	165,8	161,7-169,9
Feminino	-	-	85,3	82,1-88,4	76,5	73,5-79,5	95,2	91,6-98,8
Ambos os sexos	-	-	119,9	115,2-124,7	113,3	110,6-115,9	136,5	133,3-139,6
Atividade física acumulada								
Masculino	-	-	275,8	267,7-283,9	300,5	293,9-307,1	207,4	202,7-212,1
Feminino	-	-	184,3	176,9-191,7	204,6	199,3-209,9	109,2	105,8-112,5
Ambos os sexos	-	-	227,7	220,1-235,2	251,0	246,1-255,8	158,2	154,7-161,7

IC95%: intervalo de 95% de confiança.

* Média em atividade física expressa em minutos/semana;

** Atividade física de lazer (ou na escola, mas fora das aulas regulares).

**Tabela 3:** Média e percentis da atividade física, expressos em minutos por semana, segundo distribuição geográfica, domínios de atividade física e atividade física acumulada, conforme *Pesquisa Nacional de Saúde do Escolar* (PeNSE) de 2009, 2012, 2015 e 2019.

	Média	P10	P25	P50	P75	P90
Brasil						
PeNSE/2009	-	-	-	-	-	-
PeNSE/2012						
Educação física escolar	51,3	0	0	35	85	110
Atividade física em deslocamento ativo	58,9	0	0	35	105	175
Atividade física de lazer	122,0	0	0	50	195	455
Atividade física acumulada	232,4	15	70	175	355	540
PeNSE/2015						
Educação física escolar	50,9	0	0	45	70	130
Atividade física em deslocamento ativo	87,7	0	0	50	140	230
Atividade física de lazer	115,4	0	0	50	195	390
Atividade física acumulada	253,3	30	90	200	375	550
PeNSE/2019						
Educação física escolar	48,4	0	0	35	70	110
Atividade física em deslocamento ativo	44,5	6	6	37	44	108
Atividade física de lazer	138,5	0	25	85	210	350
Atividade física acumulada	159,2	6	37	103	231	411
Capitais						
PeNSE/2009						
Educação física escolar	57,6	0	0	45	90	135
Atividade física em deslocamento ativo	85,2	0	0	50	150	250
Atividade física de lazer	179,9	0	0	90	295	480
Atividade física acumulada	318,4	45	110	250	475	695
PeNSE/2012						
Educação física escolar	57,4	0	0	45	90	135
Atividade física em deslocamento ativo	62,0	0	0	35	105	175
Atividade física de lazer	128,8	0	0	65	195	455
Atividade física acumulada	284,4	25	85	195	380	550
PeNSE/2015						
Educação física escolar	54,1	0	0	45	90	130
Atividade física em deslocamento ativo	85,3	0	0	50	140	230
Atividade física de lazer	122,3	0	0	65	195	390
Atividade física acumulada	260,9	35	100	215	385	545
PeNSE/2019						
Educação física escolar	50,7	0	0	45	85	110
Atividade física em deslocamento ativo	46,1	6	6	37	44	111
Atividade física de lazer	145,3	5	30	100	220	385
Atividade física acumulada	162,7	6	37	107	235	416
Interior						
PeNSE/2009	-	-	-	-	-	-
PeNSE/2012						
Educação física escolar	49,4	0	0	30	85	110
Atividade física em deslocamento ativo	58,1	0	0	35	105	175
Atividade física de lazer	119,9	0	0	50	195	455
Atividade física acumulada	227,7	15	65	175	350	535
PeNSE/2015						
Educação física escolar	49,9	0	0	35	70	125
Atividade física em deslocamento ativo	88,5	0	0	50	140	230
Atividade física de lazer	113,3	0	0	45	195	390
Atividade física acumulada	251,0	30	85	195	375	550
PeNSE/2019						
Educação física escolar	47,7	0	0	35	70	110
Atividade física em deslocamento ativo	44,1	6	6	37	44	93
Atividade física de lazer	136,5	0	25	75	210	350
Atividade física acumulada	158,2	6	36	100	230	410

Realizamos a regressão de Poisson (Tabela 4) para compreendermos a associação entre o percentual de ativos fisicamente e as variáveis demográficas (sexo, idade e cor da pele), socioeconômicas (escore de bens e serviços) e comportamentais (tempo assistindo televisão e tempo sentado).

**Tabela 4:** Regressão de Poisson * analisando a associação entre o percentual de fisicamente ativos, inquéritos e variáveis demográficas, socioeconômicas e comportamentais da *Pesquisa Nacional de Saúde do Escolar* (PeNSE) de 2009, 2012, 2015 e 2019.

	RP	IC95%	Valor de p
Inquéritos			
PeNSE/2009	1,00		
PeNSE/2012	0,76	0,75-0,77	< 0,001
PeNSE/2015	0,78	0,77-0,79	< 0,001
PeNSE/2019	0,42	0,42-0,43	< 0,001
Variáveis demográficas			
Sexo			
Masculino	1,00		
Feminino	0,53	0,53-0,54	< 0,001
Idade (anos)			
13-15	1,00		
16-17	0,93	0,92-0,95	< 0,001
Cor da pele			
Branca	1,00		
Parda	1,05	1,03-1,06	< 0,001
Amarela	1,01	1,00-1,03	0,08
Preta	1,01	0,99-1,02	0,31
Indígena	1,07	1,05-1,10	< 0,001
Variável socioeconômica			
Escore de bens e serviços			
≤ 1	1,00		
2	1,09	1,07-1,12	< 0,001
3	1,15	1,13-1,18	< 0,001
4	1,27	1,25-1,30	< 0,001
Variáveis comportamentais			
Comportamento sedentário			
Tempo assistindo televisão (horas/dia)			
< 2	1,00		
≥ 2	1,01	1,00-1,02	0,07
Tempo sentado (horas/dia)			
< 3	1,00		
≥ 3	0,95	0,94-0,96	< 0,001

IC95%: intervalo de 95% de confiança; RP: razão de prevalência.

* Modelo ajustado para sexo, idade, cor da pele, escore de bens e serviços, comportamento sedentário relacionado ao tempo assistindo televisão e comportamento sedentário relacionado ao tempo sentado.

Como consequência da seleção da amostra em conglomerado, incluímos no software estatístico Stata, versão 16 (https://www.stata.com), o efeito do delineamento amostral, utilizado pelo IBGE, para a obtenção de todas as estatísticas descritivas e de associação, considerando IC95% e valor de p ≤ 0,05 como resultados estatisticamente significantes ^21^.

### Aspectos éticos

Em cada edição da PeNSE, houve aprovação da CONEP para a realização dos inquéritos: PeNSE/2009 (registro nº 11.537); PeNSE/2012 (registro nº 16.805); PeNSE/2015 (registro nº 1.006.467); e PeNSE/2019 (parecer nº 3.249.268).

## Resultados

Foram utilizados dados de 392.922 adolescentes, na faixa etária de 13 a 17 anos, a maioria sendo do sexo feminino e pardos. A descrição das características sociodemográficas e comportamentais constam no Material Suplementar (Material Suplementar https://cadernos.ensp.fiocruz.br/static//arquivo/suppl-e00063423_6796.pdf).

**Figura 1:**
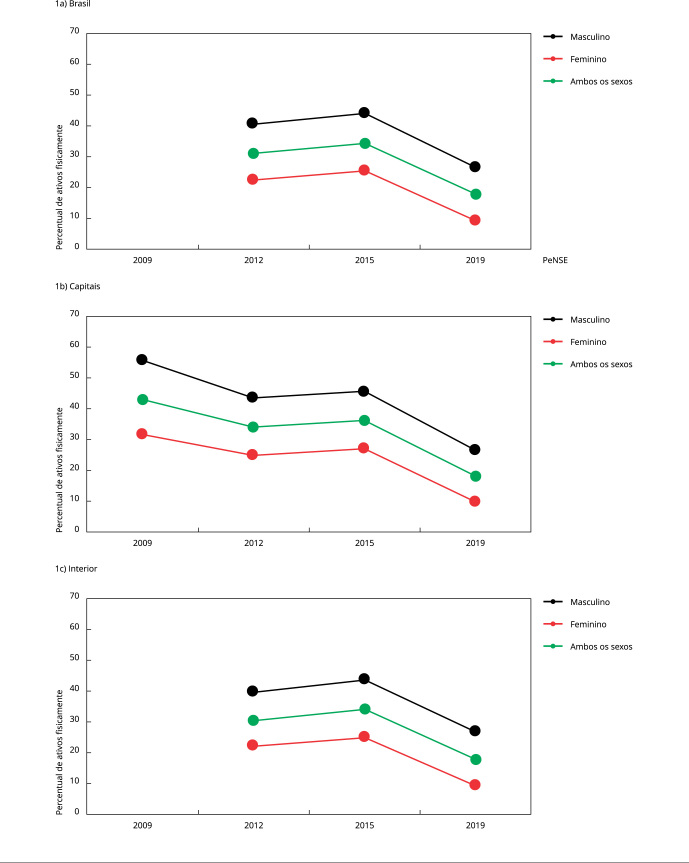
Tendência temporal do percentual de ativos fisicamente no Brasil, nas capitais e no interior nas edições da *Pesquisa Nacional de Saúde do Escolar* (PeNSE) de 2009 *, 2012, 2015 e 2019.

Há uma flutuação descendente no percentual de ativos fisicamente entre a primeira e a última edição da PeNSE, com diferenças estatisticamente significantes. Os resultados mostram que, no Brasil, o percentual de ativos fisicamente foi de 31,2% (IC95%: 30,5-32,0) na PeNSE/2012 e houve uma queda abrupta para 18% (IC95%: 17,5-18,6) na PeNSE/2019. A flutuação foi semelhante nos municípios do interior: de 30,4% (IC95%: 29,2-31,6) na PeNSE/2012 para 17,9% (IC95%: 17,3-18,6) na PeNSE/2019. A tendência de reduzir o percentual de ativos fisicamente com flutuação descendente também foi observada nas capitais: de 43,1% (IC95%: 42,2-43,9) na PeNSE/2009 para 18,2% (IC95%: 17,5-19,0) na PeNSE/2019 (Tabela 1; Figura 1).

* A PeNSE/2009 coletou dados apenas nas 27 capitais brasileiras.

A flutuação observada no percentual de ativos fisicamente também ocorreu na média em atividade física: (i) no Brasil, de 232,4 minutos/semana (IC95%: 227,7-237,0) na PeNSE/2012 para 159,2 minutos/semana (IC95%: 156,4-162,0) na PeNSE/2019; (ii) nos municípios do interior, de 227,7 minutos/semana (IC95%: 220,1-235,2) para 158,2 minutos/semana (IC95%: 154,7-161,7); e (iii) nas capitais, de 318,4 minutos/semana (IC95%: 313,4-323,4) para 162,7 minutos/semana (IC95%: 159,1-166,2). Nas capitais, ao longo de dez anos, a redução foi de 50% na média em atividade física (Tabela 2), representando o caminho inverso das diversas recomendações sobre atividade física para adolescentes.

**Figura 2:**
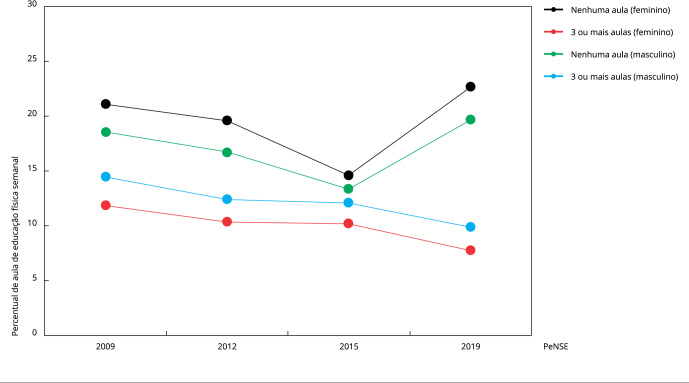
Percentual de adolescentes que não tiveram aula ou tiveram três ou mais aulas de educação física nos últimos sete dias, distribuído por sexo, conforme *Pesquisa Nacional de Saúde do Escolar* (PeNSE) de 2009 *, 2012, 2015 e 2019.

Por sexo, as meninas tiveram um percentual de ativos fisicamente menor em relação aos meninos em todas as edições, estatisticamente significante para Brasil, capitais e interior e entre inquéritos (Tabela 1). Elas também têm percentual de ativos fisicamente menor no lazer. Entretanto, na educação física escolar, apesar de haver uma redução, a variação é baixa (9 minutos/semana) entre os inquéritos, mesmo que o tempo de atividade física das meninas seja inferior a 50 minutos/semana e o dos meninos seja superior a 60 minutos/semana (Tabela 2). Isso contribui para que o percentual de ativos fisicamente das meninas seja 47% menor que o dos meninos, conforme regressão de Poisson (Tabela 4). Ou seja, quando há autorrelato de não ter tido aulas de educação física, as meninas têm maior prevalência; e quando os escolares referem ter tido três ou mais aulas, os meninos têm percentual maior de participação (Figura 2).

* A PeNSE/2009 coletou dados apenas nas 27 capitais brasileiras.

O escore socioeconômico revela que são fisicamente ativos, com diferença estatisticamente significante, aqueles que têm todos os bens do escore, sendo que em 2012 e 2015 essa diferença ocorreu de forma progressiva nos quatro níveis de posse (Tabela 1). Não houve diferença entre as duas faixas etárias (13-15 anos e 16-17 anos).

Quando comparamos o percentual de ativos fisicamente por meio do IC95% entre municípios das capitais e do interior, encontramos diferença estatisticamente significativa nas PeNSE/2012 e PeNSE/2015. Nas capitais e no interior, há diferença estatística entre os inquéritos (Tabela 1).

Nossas análises revelam que a variação no deslocamento, aferida pela mediana, foi de 15 minutos entre 2009 e 2019. Porém, 90% (P90) dos adolescentes em 2009 não atingiam as recomendações de atividade física de intensidade moderada à vigorosa nesse domínio, mas eram mais ativos (250 minutos/semana) do que foram os de 2019 (108 minutos/semana), conforme Tabela 3. Em média, a diferença da PeNSE/2019 quanto ao deslocamento, em relação aos inquéritos anteriores, é estatisticamente significativa (Tabela 2). A atividade física no lazer também é estatisticamente significativa entre todos os inquéritos e mantém comportamento decrescente, com redução, em média, de 40 minutos, mesmo que em 2019 tenha ocorrido um aumento em relação a 2012 e 2015 (Tabela 2).

A mudança no comportamento sedentário foi estatisticamente significativa nos acompanhados por meio do tempo assistindo televisão e tempo sentado. Assistir TV por mais de duas horas por dia foi informado por 79,8% (IC95%: 79,2-80,4) em 2009 e por 46% (IC95%: 45,1-46,9) em 2019, revelando uma redução de 50%. Ter o hábito de ficar sentado (tempo sentado) por mais de três horas saltou de 50,1% (IC95%: 48,9-51,3) em 2009 para 54% (IC95%: 53,1-54,9) em 2019.

## Discussão

Com relação ao Brasil, houve redução do percentual de ativos fisicamente (13,9% entre meninos e 13,3% entre meninas) no período de 2012 a 2019 (Figura 1), aproximando-se da prevalência encontrada por Ferrari et al. ^22^ relativa a atingir a recomendação da OMS ^13^^,^^19^ de praticar atividade física por tempo igual ou superior a 60 minutos/dia. Entretanto, nas capitais, a queda foi vertiginosa na década estudada, pois o percentual de meninos ativos fisicamente recrudesceu 29,3%, e de meninas, 21,8%, de maneira que, ao fim do período, os ativos fisicamente representaram menos de 30% (meninos) e 10% (meninas) (Figura 1).

Tais reduções são reforçadas com a questão de não ter tido aula de educação física na última semana, pois essa afirmação esteve mais presente no autorrelato de meninos (+1,1%) e meninas (+1,6%). Concomitantemente, ter tido três ou mais aulas foi menos relatado por meninos (-4,5%) e meninas (-4,1%) (Figura 2).

Um dos caminhos a seguir é, indubitavelmente, promover a atividade física no cotidiano das pessoas, sobretudo de crianças e adolescentes, sendo uma forma eficiente para enfrentar a pandemia da inatividade física ^23^, com seus altos percentuais de prevalência na população - adultos ^20^^,^^23^^,^^24^, crianças e adolescentes ^9^^,^^10^^,^^12^^,^^25^^,^^26^. Assim, é possível consolidar hábitos saudáveis antes dos padrões comportamentais se tornarem resistentes às mudanças.

Nesse contexto, a escola pode contribuir para elevar o percentual de ativos fisicamente e reduzir a inatividade física. Tal assertiva encontra respaldo nos benefícios gerados pela atividade física na vida dos indivíduos, que incluem melhoria dos resultados acadêmicos ^27^^,^^28^^,^^29^, redução dos riscos cardiovasculares ^29^^,^^30^^,^^31^, melhoria da saúde musculoesquelética ^30^^,^^32^ e dos perfis lipídico e metabólico ^33^.

Infelizmente, nos últimos seis anos, o Brasil foi na contramão do mundo, reduzindo a prática de educação física escolar a uma aula por semana em muitas redes de ensino. Isso afeta a quantidade de estímulos aos quais os adolescentes estão expostos e os afasta da prática de atividade física fora da escola. Ou seja, a educação física escolar não tem o objetivo de tornar os adolescentes exaustivamente ativos durante a aula, mas sim expô-los à cultura corporal do movimento e, nesse cenário, incentivar a realização de atividades físicas em outros contextos.

Porém, é necessário reverter o número total de aulas semanais ofertadas - apenas 11,3% (PeNSE/2015) ^26^ e 8,9% (PeNSE/2019) ^10^ dos adolescentes brasileiros tiveram três ou mais aulas de educação física nos últimos sete dias ^9^^,^^25^. Em estudo anterior, foi demonstrado que há forte correlação (respectivamente, rh0 = -0,84 e rh0 = -0,81) entre não ter tido aula de educação física nos últimos sete dias e o percentual de escolares ativos ^34^, tanto na PeNSE/2009 quanto na PeNSE/2012.

Nossa análise mostra que há, na educação física escolar, diferença estatisticamente significante entre a PeNSE/2009 (57,6 minutos/semana; IC95%: 55,2-60,0) e a PeNSE/2019 (48,4 minutos/semana; IC95%: 46,9-49,8). A análise também reporta diferença na participação das meninas nas aulas desse componente curricular, que estão, aproximadamente, 20 minutos aquém da prática realizada pelos meninos em todos os inquéritos no Brasil, nas capitais e no interior - em percentual de ativos fisicamente (Tabela 1), em média de atividade física (Tabela 2) e reforçada pela regressão de Poisson (Tabela 4).

A atividade física mostra uma grande variação no domínio do lazer. Entre 2012 e 2019, em média, houve aumento de 15,1 minutos/semana nas capitais e 9,9 minutos/semana no interior entre as meninas, enquanto os meninos tiveram incremento de 4,3 minutos/semana nas capitais e 7,4 minutos/semana no interior (Tabela 2). Entretanto, quando analisamos apenas as capitais, de 2009 a 2019, houve um decréscimo de 6,9 minutos/semana entre as meninas e de alarmantes 81,5 minutos/semana entre meninos.

Sabe-se que o lazer envolve a existência de praças e parques próximos da residência, instalações adequadas e condições seguras para deslocamento (violência da região, iluminação), mas o que justifica a atividade física das meninas, na educação física escolar, ser cerca de 21 minutos/semana mais breve do que a dos meninos? A educação física escolar não é um lugar democrático, inclusivo, acessível a todos, partilhado e, principalmente, pedagogicamente orientado?

Se considerarmos que todas as aulas foram ministradas, que as 28 semanas letivas na educação brasileira transcorreram normalmente, a diferença encontrada representa que, de 13 a 17 anos, as meninas perderam, em média, 49 horas de aulas de educação física - exatamente no período em que poderiam ter uma aula sobre e com o movimento corporal igual a de seus pares, desenvolver habilidades motoras e adquirir conhecimento e hábito da atividade física para toda a vida.

Marramarco ^35^ analisou o desenvolvimento de habilidades motoras de uma população que apresentava fatores ambientais favoráveis ao pleno desenvolvimento (assistência à saúde, alimentação adequada e condições de higiene), baixo índice de desnutrição e de preocupação em relação ao sobrepeso e à obesidade. Todavia, mesmo em condições propícias, as meninas apresentaram resultados inferiores aos dos meninos, tanto nas habilidades de locomoção como nas capacidades de controle de objeto e no coeficiente de motricidade ampla.

Não é de nosso interesse dissertar sobre a puberdade e as alterações típicas dessa etapa da vida dos indivíduos. Entretanto, alguns aspectos precisam ser elencados, já que, nas aulas de educação física, a exclusão se dá pelo grau de habilidade e força dos participantes. A força começa a se diferenciar na puberdade, que pode ocorrer entre 8 e 13 anos de idade nas meninas.

A habilidade motora fundamental inclui as capacidades motoras básicas (força, velocidade e resistência), necessárias para tarefas específicas do movimento, e precisa ser lapidada para promover o desenvolvimento motor. Experimentar-se motoramente fornece uma abundância de informações e percepções sobre si mesma e sobre o mundo que a cerca, contribuindo para que a criança se desenvolva cognitiva e fisicamente, progrida sequencialmente de um estágio a outro, influenciada pelo amadurecimento e pelo conhecimento. Não se trata exclusivamente da maturação, mas sim de oportunidades de prática, encorajamento e instruções, que são cruciais para o desenvolvimento de padrões maduros de movimentos fundamentais ^36^.

Encontramos respaldo em estudo desenvolvido por Kremer et al. ^37^, que utilizaram acelerometria para mensurar a intensidade e duração dos esforços físicos nas aulas de educação física, concluindo que os meninos (44,1%) estiveram em atividade física de intensidade moderada à vigorosa por mais tempo que as meninas (21%; p < 0,01). Fortes et al. ^38^ concluíram que a maioria das aulas são definidas como “aula livre”, o esporte é o conteúdo prioritário das aulas e a ênfase estava na ação dos professores sob a forma de observação e/ou outras tarefas. Soma-se a isso o declínio acentuado no percentual de ativos fisicamente que ocorre na adolescência quando comparado à infância ^39^. Estamos diante de uma situação complexa, que precisa de políticas públicas, formações e orientações que conduzam à reversão da inatividade física.

Contudo, estudo realizado por Ferrari et al. ^22^ mostrou que o ambiente escolar está associado ao aumento da atividade física de diversas maneiras: (i) associação positiva entre a atividade física total - obtida somando todos os domínios da atividade física - e a existência de infraestrutura nas escolas: quadras poliesportivas (três ou mais), piscina em condições de uso, pistas de corrida/atletismo e bicicletários; (ii) infraestrutura na escola e no entorno (quadras poliesportivas, piscina disponível em condições de uso, semáforos com limite de velocidade ao redor da escola e faixas de pedestres) está positivamente associada às aulas de educação física; e (iii) a existência de bicicletários, de faixas de pedestres e a limitação de velocidade ao redor da escola foram positivamente associadas ao deslocamento ativo.

Assim, é importante fortalecer o sistema de monitoramento da atividade física dos adolescentes, que representa uma forma eficaz de analisar os efeitos de diferentes estímulos, resultantes dos exercícios físicos (movimento corporal planejado, organizado e repetitivo) ^40^ e de outras formas de atividade física (por exemplo, deslocamentos para a escola). Esse sistema também permite compreender como as formas institucionalizadas estão sendo ofertadas aos adolescentes (por exemplo, educação física escolar ou políticas públicas esportivas) e, inclusive, acompanhar o desenvolvimento da pandemia de inatividade física, que conduz a diversos desfechos em saúde, entre eles a obesidade, DCNT que gera consequências na idade adulta, a curto e longo prazo, incluindo mortalidade precoce e morbidade física.

Adolescentes fisicamente ativos aumentam a probabilidade de serem adultos ativos, contribuindo para o balanço energético (consumo e gasto), reduzem a probabilidade de desenvolver obesidade e doenças relacionadas à obesidade na fase adulta e, o mais importante, equilibram o balanço energético durante a adolescência, pois estão protagonizando a prevenção e a profilaxia da obesidade e de doenças relacionadas nessa fase do ciclo vital.

Com relação ao comportamento sedentário, sabe-se que há pouco mais de 20 anos ele foi reconhecido como problema de saúde pública e, ao mesmo tempo, é um modulador das taxas de prevalência das DCNT. Isso ocorre porque o comportamento sedentário influencia a redução do percentual de ativos fisicamente, somado à facilidade que os indivíduos têm de usufruir das benesses das novas tecnologias (assistir TV, jogar videogames, navegar pela Internet, por exemplo), ao mesmo tempo que as formas de trabalho, baseadas na força física, foram abrandadas com as revoluções industrial e tecnológica, sendo substituídas pelo maior tempo despendido em trabalho intelectual - normalmente, tempo sentado.

A maneira como se mensura o comportamento sedentário ainda precisa evoluir para que se possa afirmar com exatidão se ações caracterizadas como sedentárias contribuem ou não para que os indivíduos usufruam das benesses que a sociedade conquistou ao longo dos últimos séculos sem se expor às DCNT. É especialmente importante entender que há videogames que exigem a execução de movimentos pelo praticante, portanto, reduzindo o impacto de uma atividade que é considerada prejudicial à saúde dos indivíduos e, quiçá, revertendo os efeitos deletérios do tempo adicional sentado.

Considera-se como limitação neste estudo a análise da PeNSE/2009, pois refere-se exclusivamente às 27 capitais brasileiras, sem aferição da atividade física e demais condições de saúde dos adolescentes no interior. Assim, a tendência temporal das capitais inclui as quatro edições da PeNSE (2009, 2012, 2015 e 2019), mas o interior do Brasil está restrito às edições de 2012, 2015 e 2019. Consequentemente, a análise de Brasil também exclui a PeNSE/2009.

## Conclusão

Em nossos achados, dois aspectos merecem atenção redobrada dos gestores do Ministério da Saúde e das Secretarias Estaduais e Municipais de Saúde: a queda abrupta da prevalência de atividade física e o resultado da inequidade entre os sexos no percentual de ativos fisicamente.

Primeiro, a média em atividade física regrediu de 318,4 minutos/semana (2009) para 159,2 minutos/semana, queda de aproximadamente 50% em dez anos, e o Brasil está no nível de países considerados desenvolvidos, como demonstrado no resultado do HBSC. Porém, tal processo foi progressivo e os gestores públicos tiveram oportunidades de rever as ações de promoção da atividade física em 2012 (232,4 minutos/semana) ou em 2015 (253,3 minutos/semana), pois uma parcela significativa já não estava cumprindo as recomendações de 300 minutos/semana em atividade física de intensidade moderada à vigorosa. O segundo aspecto é o resultado entre sexos nas aulas de educação física, pois as meninas estão, em média, 21 minutos/semana aquém do tempo de prática dos meninos nesse domínio.

É imprescindível que o número de aulas seja ampliado para, no mínimo, três vezes na semana, e que os escolares sejam estimulados à prática de atividade física fora do ambiente escolar. Além disso, mudanças metodológicas, didaticamente conscientes e pedagogicamente igualitárias, devem ser conduzidas, pois parece inaceitável querermos promover a interação entre meninas e meninos se elas são preteridas nas aulas de educação física, muito provavelmente em razão das habilidades motoras dos meninos, que são, em nossa sociedade, estimulados ao esporte, à aventura, ao lúdico em ambientes abertos, enquanto as meninas fantasiam a vida de donas do lar. Ampliamos a lacuna no desenvolvimento motor ao considerarmos que todas as questões culturais desaparecem quando meninas e meninos se juntam na quadra/pátio/ginásio da escola e não percebemos que três horas por semana não impedirão o desenvolvimento social e afetivo dos adolescentes. Portanto, é provável que a solução seja separar as turmas por sexo e permitir que as meninas se apropriem da cultura corporal do movimento e desenvolvam habilidades motoras que lhes têm sido negadas ao longo dos anos.
