# Supplementary material for: Temporal trend of physical activity in Brazilian adolescents: analysis of the Brazilian National Survey of School Health from 2009 to 2019
Source: Cad Saude Publica. 2023 Nov 13;39(10):e00063423. [Article in Portuguese] doi: 10.1590/0102-311XPT063423 (PMC10652710; doi:10.1590/0102-311XPT063423)
Supplement: Supplementary file 1 [file 1678-4464-csp-39-10-PT063423-s.pdf]

MATERIAL SUPLEMENTAR

Distribuição da amostra segundo variáveis demográficas, socioeconômicas e comportamentais da *Pesquisa Nacional de Saúde do Escolar* (PeNSE) de 2009, 2012, 2015 e 2019.

**Variáveis**	**PeNSE**							
	**2009 (n = 61.434)**		**2012 (n = 106.480)**		**2015 (n = 100.110)**		**2019 (n = 124.898)**	
	**%**	**IC95%**	**%**	**IC95%**	**%**	**IC95%**	**%**	**IC95%**
Sexo								
Masculino	47,5	46,8-48,2	47,8	45,8-49,8	48,6	48,0-49,2	49,3	48,5-50,1
Feminino	52,5	51,8-53,2	52,2	50,2-54,2	51,4	50,8-52,0	50,7	49,9-51,5
Idade (anos)								
13-15	90,6	89,8-91,4	88,1	83,0-91,9	90,2	89,6-90,7	64,7	62,4-66,9
16-17	9,4	8,6-10,2	11,9	8,1-17,0	9,8	9,2-10,4	35,3	33,1-37,6
Cor da pele								
Branca	40,2	38,7-41,9	37,0	31,9-42,4	36,3	35,2-37,4	36,0	35,1-36,8
Parda	39,1	37,8-40,4	42,1	39,0-45,4	43,1	42,1-44,0	43,5	42,7-44,4
Amarela	3,7	3,4-4,1	4,1	3,4-4,9	4,1	3,9-4,4	3,7	3,5-4,0
Preta	12,8	12,1-13,5	13,2	11,2-15,6	13,2	12,6-13,7	13,6	13,1-14,1
Indígena	4,1	3,8-4,4	3,5	2,8-4,4	3,3	3,1-3,5	3,1	2,9-3,4
Escore de bens e serviços *								
≤ 1	13,8	12,9-14,7	9,6	8,4-11,0	6,6	6,1-7,1	6,4	5,9-7,0
2	24,9	23,6-26,1	22,6	21,6-23,7	12,6	12,0-13,1	11,2	10,7-11,8
3	17,5	16,7-18,4	16,1	15,2-16,9	21,8	21,1-22,5	32,9	32,1-33,7
4	43,8	41,6-46,1	51,7	50,1-53,2	59,0	57,8-60,2	49,5	48,2-50,7
Comportamento sedentário relacionado ao tempo assistindo televisão (horas/dia)								
< 2	20,2	19,6-20,8	21,7	21,1-22,3	40,1	39,2-41,0	63,7	62,9-64,5
≥ 2	79,8	79,2-80,4	78,3	77,7-78,8	59,9	59,0-60,8	36,3	35,5-37,1
Comportamento sedentário relacionado ao tempo sentado (horas/dia)								
< 3	49,9	48,7-51,1	37,6	36,6-38,7	43,4	42,7-44,2	46,0	45,1-46,9
≥ 3	50,1	48,9-51,3	62,4	61,3-63,4	56,6	55,8-57,3	54,0	53,1-54,9

IC95%: intervalo de 95% de confiança.

* Criado a partir da posse de celular, computador, Internet e banheiro em casa.
